# Gastric emptying performance of stomach-partitioning gastrojejunostomy *versus* conventional gastrojejunostomy for treating gastric outlet obstruction: A retrospective clinical and numerical simulation study

**DOI:** 10.3389/fbioe.2023.1109295

**Published:** 2023-02-17

**Authors:** Haiqiao Zhang, Fengyan Xu, Zhi Zheng, Xiaoye Liu, Jie Yin, Zhenmin Fan, Jun Zhang

**Affiliations:** ^1^ Department of General Surgery, Beijing Friendship Hospital, Capital Medical University, Beijing, China; ^2^ School of Mechanical Engineering, Jiangsu University of Technology, Changzhou, Jiangsu, China

**Keywords:** gastric outlet obstruction, delayed gastric emptying, stomach-partitioning gastrojejunostomy, conventional gastrojejunostomy, numerical simulation

## Abstract

**Purpose:** This study evaluated the gastric emptying performance of stomach-partitioning gastrojejunostomy (SPGJ) *versus* conventional gastrojejunostomy (CGJ) for treating gastric outlet obstruction (GOO).

**Methods:** First, 73 patients who underwent SPGJ (n = 48) or CGJ (n = 25) were involved. Surgical outcomes, postoperative recovery of gastrointestinal function, delayed gastric emptying, and nutritional status of both groups were compared. Second, a three-dimensional stomach model was constructed based on the gastric filling CT images from a GOO patient with a standard stature. The present study evaluated SPGJ numerically by comparing it with CGJ in terms of local flow parameters such as flow velocity, pressure, particle retention time, and particle retention velocity.

**Results:** Clinical data found that SPGJ had significant advantages over CGJ in terms of time to pass gas (3 *versus* 4 days, *p* < 0.001), time to oral intake (3 *versus* 4 days, *p* = 0.001), postoperative hospitalization (7 *versus* 9 days, *p* < 0.001), the incidence of delay gastric emptying (DGE) (2.1% *versus* 36%, *p* < 0.001), DGE grading (*p* < 0.001), and complications (*p* < 0.001) for GOO patients. Moreover, numerical simulation revealed that the SPGJ model would induce contents in stomach discharge to the anastomosis at a higher speed, and only 5% of that flowed to the pylorus. SPGJ model also had a low-pressure drop as the flow from the lower esophagus to the jejunum, reducing the resistance to food discharge. Besides, the average retention time of particles in the CGJ model is 1.5 times longer than that in the SPGJ models, and the average instantaneous velocity in CGJ and SPGJ models are 22 mm/s and 29 mm/s, respectively.

**Conclusion:** Compared with CGJ, patients after SPGJ had better gastric emptying performance and better postoperative clinical efficacy. Therefore, we think that SPGJ may be a better option for treating GOO.

## Introduction

Gastric outlet obstruction (GOO) is a digestive system disease of pyloric and duodenal stenosis mainly caused by gastric cancer, duodenal ulcer, and pancreatic cancer (Chowdhury et al., 1996). In recent years, applying proton pump inhibitors have greatly reduced the occurrence of peptic ulcers (Feinstein et al., 2010). Therefore, 50%–80% of GOO are caused by malignant tumors (Appasani et al., 2011). Among them, pancreatic cancer is the most common cause in Western countries, while gastric cancer is the leading cause in Asia (Troncone et al., 2020). The main focus of treatment for GOO is to resume oral intake. Treatments include surgery and endoscopic stent placement. Although stent placement can avoid the trauma caused by surgery, obstruction is prone to recurrence (Jue et al., 2021). The surgical treatments for GOO mainly include conventional gastrojejunostomy (CGJ) and stomach-partitioning gastrojejunostomy (SPGJ) (Kaminishi et al., 1997). CGJ is simple and widely used, but this treatment could delay gastric emptying frequently after surgery (Kumagai et al., 2016; Lorusso et al., 2019). In 1997, SPGJ was first reported by Kaminishi (Kaminishi et al., 1997). It partially separates the stomach, which is conducive to food emptying, and effectively reduces food stimulation of the tumor. At present, several studies have shown that SPGJ has a significant advantage over CGJ in terms of delayed gastric emptying (DGE) (Table 1) (Kaminishi et al., 1997; Kato et al., 2001; Yamagishi et al., 2004; Oida et al., 2009; Usuba et al., 2011; Abdel-lah-Fernandez et al., 2015; Ernberg et al., 2015; Kumagai et al., 2016; Lorusso et al., 2019; Yildirim et al., 2020). Meanwhile, SPGJ has advantages over CGJ in terms of gastrointestinal function recovery, the proportion of oral intake, length of stay, and survival after surgery. And it does not increase operative time, intraoperative blood loss, or anastomotic complications. However, the efficacy advantages of SPGJ are still lacking in effective persuasion. A meta-analysis (Lorusso et al., 2019) of 226 GOO patients from 8 studies showed that the incidence of DGE was 43.6% (27/62) after CGJ and 11.6% (8/69) after SPGJ. In the CGJ and SPGJ groups, 56.5% (26/46) and 89.6% (43/48) of patients returned to oral intake after surgery, respectively. However, the sample size of the included studies in this meta-analysis was small, all of which were retrospective studies, and the enrolled patients had different pathological characteristics. In addition, these studies lacked the unified definition and classification of DGE, leading to differences in evaluation, which may lead to results bias.

**TABLE 1:** Characteristics of included studies.

Author	Year	Group	N	DGE, n (%)	Grade A/B/C	*p*-Value
Kaminishi (Kaminishi et al., 1997)	1997	SPGJ	8	1 (12.5)	—	0.038^a^
		CGJ	13	9 (69.2)	—	
Kato (Kato et al., 2001)	2001	SPGJ	7	1 (14.3)	—	0.735
		CGJ	5	2 (40)	—	
Yamagishi (Yamagishi et al., 2004)	2004	SPGJ	4	0 (0)	—	0.279
		CGJ	8	4 (50)	—	
Oida (Oida et al., 2009)	2009	SPGJ	30	2 (6.7)	—	<0.001^a^
		CGJ	30	14 (46.7)	—	
Usuba (Usuba et al., 2011)	2011	SPGJ	26	6 (23.1)	—	0.216
		CGJ	20	8 (40)	—	
Ernberg (Ernberg et al., 2015)	2015	SPGJ	10	5 (50)	5/0/0	0.780
		CGJ	14	9 (64.3)	3/1/5	
Abdel-lah-Fernandez (Abdel-lah-Fernandez et al., 2015)	2015	SPGJ	7	1 (7.1)	—	0.771
		CGJ	9	3 (33.3)	—	
Yildirim (Yildirim et al., 2020)	2020	SPGJ	16	3 (18.75)	3/0/0	0.001^a^
		CGJ	37	26 (70.3)	16/4/6	

^a^ Statistically significant values.

SPGJ, stomach-partitioning gastrojejunostomy; CGJ, conventional gastrojejunostomy; N, number of the patients; DGE, delayed gastric emptying.

Furthermore, the dynamic mechanism of SPGJ and CGJ in gastric emptying is currently unclear. The mechanisms involved in gastric emptying are complex and multifactorial. Nevertheless, the physical properties and geometry of the upper digestive tract motion play a critical role in gastric emptying. Moreover, studies revealed that flow field and the mechanical pattern had a great relationship with the mixing, flow, and emptying of food or drugs in the stomach (Pal et al., 2004; Ferrua and Singh, 2010; Acharya et al., 2021; Li et al., 2021; Seo and Mittal, 2021). Pal constructed an actual gastric geometric model based on magnetic resonance imaging and analyzed the effects of antral contraction waves on the movement, pressure and mixing of content through computational fluid dynamics (Pal et al., 2004). This study is the first to apply 3D models and numerical simulations to gastric flow, generating new data not available in previous experiments and advancing our understanding of contents emptying. Ferrua analyzed the hydrodynamics of contents with different viscosities through a three-dimensional (3D) model of the stomach. And he found that the most vigorous fluid movement was in the antrum, and identified a vital recirculation of contents from the bottom to the antrum (Ferrua and Singh, 2010). Therefore, the 3D model combined with the numerical simulation method can analyze the upper digestive tract hydrodynamics objectively. We believe flow patterns after different surgery for GOO may lead to different gastric functions, hence suppressing or worsening the adverse events after surgical treatment.

To verify our hypotheses, the present study retrospectively analyzed the efficacy of SPGJ and CGJ for treating GOO, and 3D computational fluid dynamics models based on the patient-specific stomach were investigated for the flow performance, providing insight into the treatment for GOO.

## Methods

### Study population

Clinical data of GOO patients admitted to Affiliated Beijing Friendship Hospital, Capital Medical University from January 2015 to March 2022, were retrospectively analyzed in this trial. The diseases were determined *via* gastroscopic and abdominal-enhanced computed tomography (CT). Inclusion criteria were as follows: cT_1–4_N_−/+_M_1_ lower gastric cancer or benign pyloric obstruction, aged 18–75 years, with no sex restrictions, performed SPGJ or CGJ, with or without conversion to laparotomy, no cerebrovascular injury or severe heart disease, no history of epilepsy, central nervous system disease or mental illness, no organ transplantation, no pregnancy or lactation, and complete case information. From January 2015 to July 2018, the surgeon mainly treated patients with CGJ. From August 2018 to March 2022, the surgeon mainly treated patients with SPGJ.

### Surgical techniques

**FIGURE 1:**
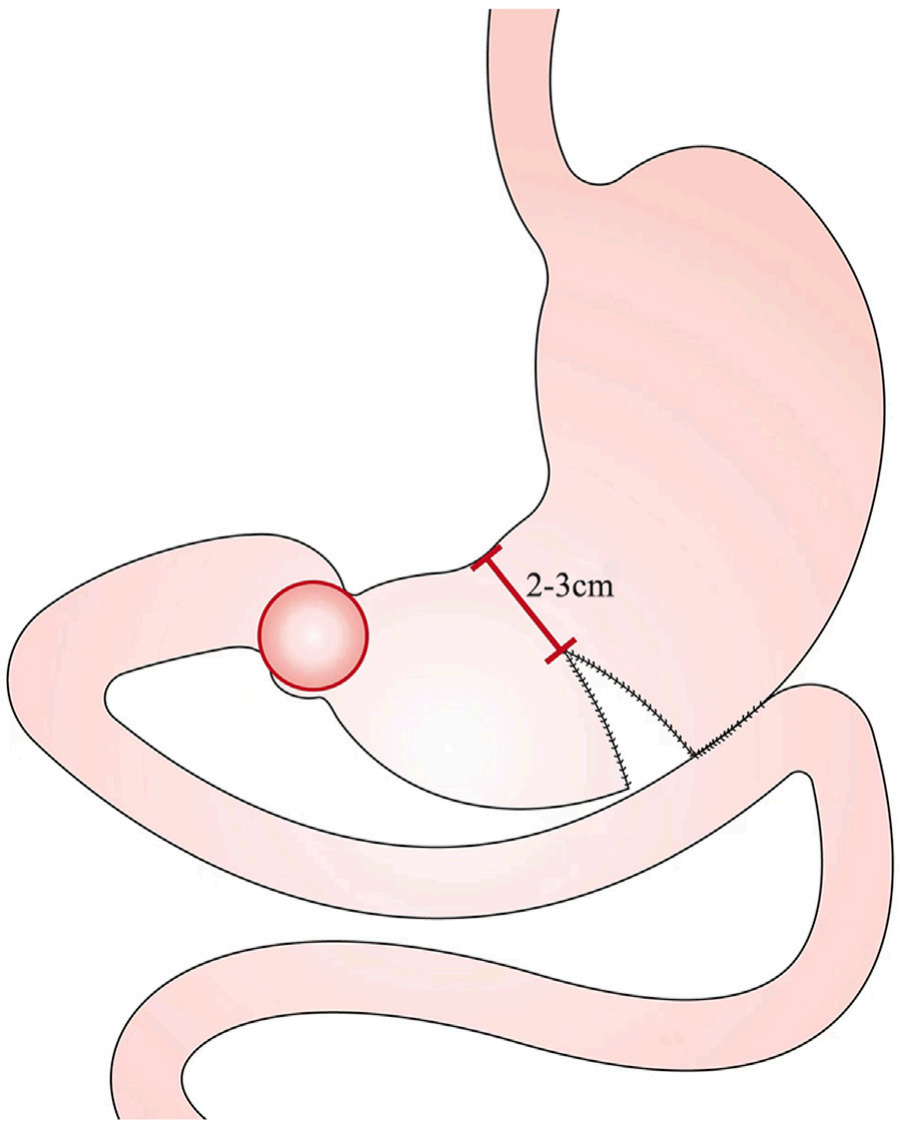
The schematic diagram of SPGJ.

The SPGJ procedure is illustrated in Figure 1. A separate part of the stomach at the junction of the gastric corpus and antrum or about 5 cm from the upper edge of the tumor with a linear stapler, leaving the 2–3 cm wide gastric corpus near the lesser curvature. Cut an opening at the margin of the greater curvature of the proximal stomach. Cut another opening on the opposing mesangial limbus of the jejunum 5–10 cm away from the ligament of flexion. Then a linear stapler is inserted, and the greater curvature and the jejunum are side-to-side anastomosed at the back of the colon. The opening was closed by continuous suture with barbed wire.

**FIGURE 2:**
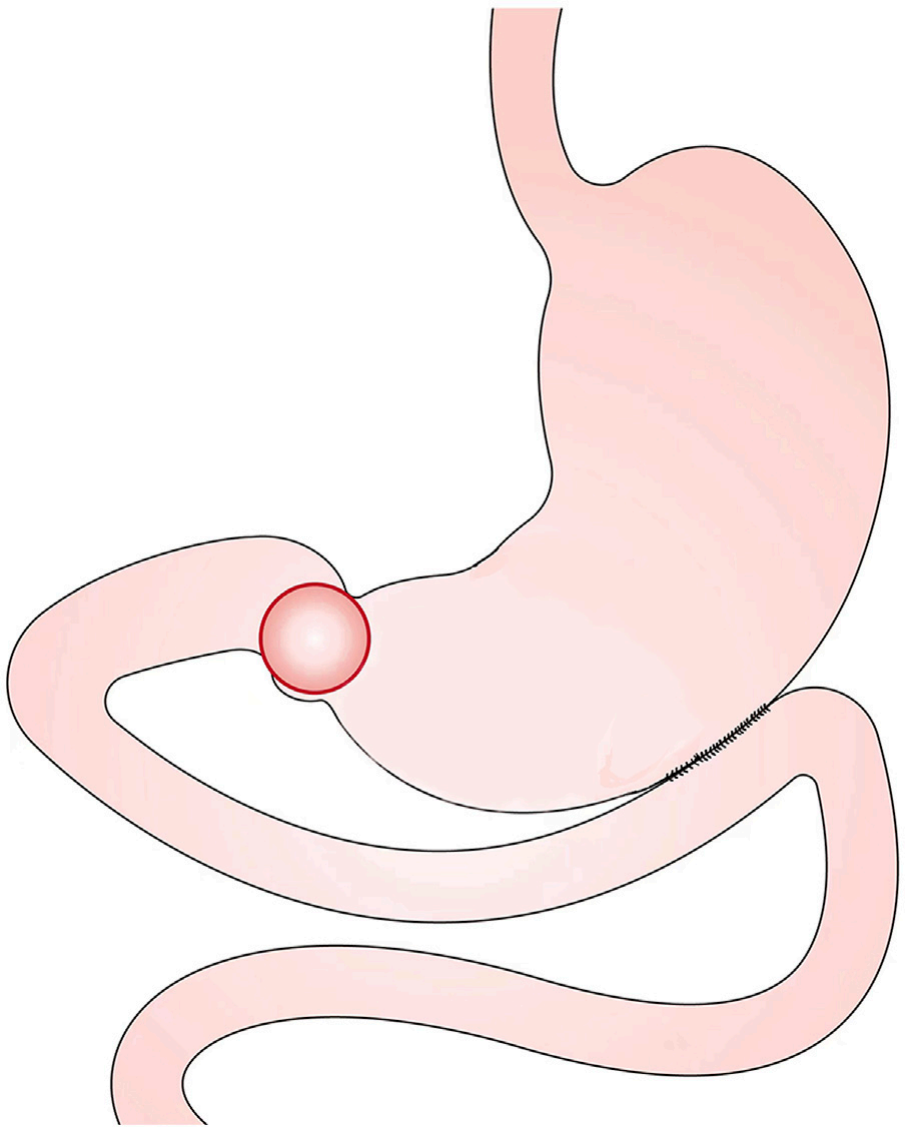
The schematic diagram of CGJ.

The CGJ procedure is illustrated in Figure 2. Cut an opening at the lowermost point of the greater curvature or about 5 cm from the upper edge of the tumor. Cut another opening on the opposing mesangial limbus of the jejunum 5–10 cm away from the ligament of flexion. Then a linear stapler is inserted, and the greater curvature and the jejunum are side-to-side anastomosed at the back of the colon. The opening was closed by continuous suture with barbed wire.

### Perioperative outcomes and follow-up

Study outcomes assessed included operative time, blood loss, time to pass gas, time to oral intake, postoperative hospitalization days, complications, incidence and grade of DGE, and postoperative nutritional status. The nutritional status was assessed using the gastric outlet obstruction scoring system (GOOSS): 0 = no oral intake, 1 = liquids only, 2 = soft solids, 3 = low-residue or full diet. Preoperative nutritional assessment according to Controlling Nutritional Status (COUNT) standard (Kuroda et al., 2018). CONUT is calculated as the sum of the serum albumin score, total lymphocyte score, and total cholesterol score. DGE grade according to ISGPS grading standard (Wente et al., 2007). Postoperative complications were classified according to the Clavien-Dindo (CD) standard (Dindo et al., 2004).

### Statistical analysis

Statistical analyses were performed using SPSS 21.0. Normally distributed data were expressed as a mean ± standard deviation, and values were compared using the independent sample *t*-test. Non-normally distributed data were expressed as median (interquartile range), and values were compared using the non-parametric test. A non-parametric test was used to compare the grade data. *p* < 0.05 is set as the significance level.

### The geometry models

**FIGURE 3:**
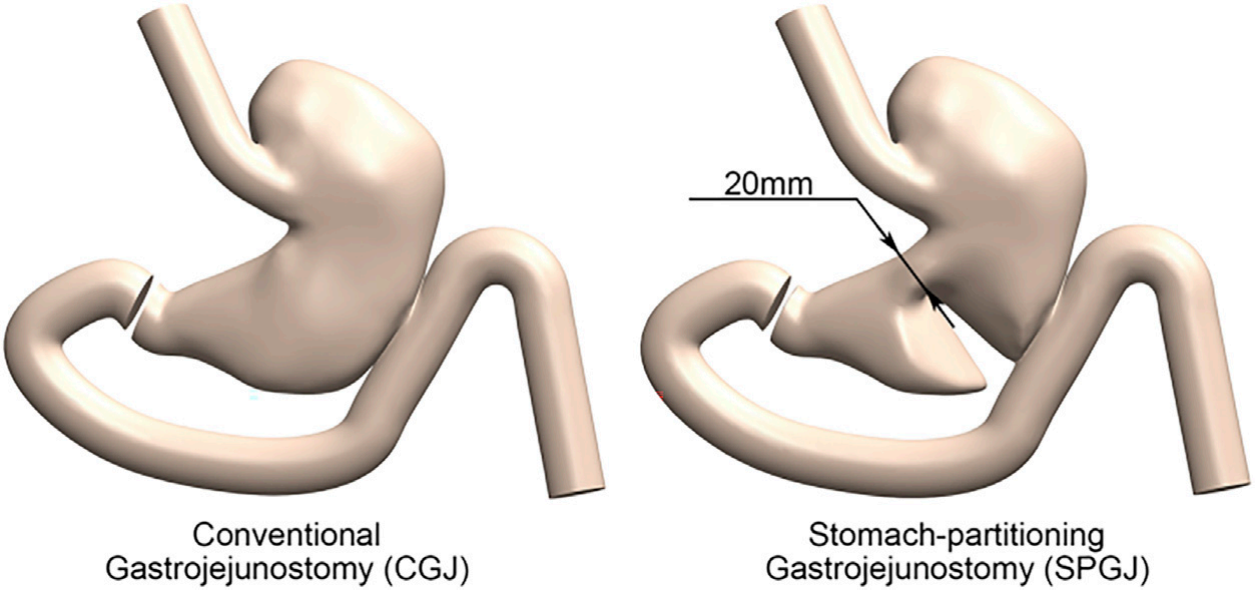
The 3D computational model of CGJ and SPGJ used in numerical simulation.

A male GOO patient with a BMI of 22.15 kg/m^2^ was selected to construct a three-dimensional model of the upper digestive tract with gastric filling phase abdominal CT. The gastric images in this study included the stomach, esophagus, duodenum, and jejunum. In our study, a total of 96 contiguous slices were captured by an imager (GE_MEDICAL_SYSTEMS/Revolution CT), generated with a 512 × 512-pixel image size, and a 0.7 mm pixel size. The lumen boundaries of the stomach were manually segmented by Mimics 10.0 (Materialise N.V.) to develop the gastric model. This three-dimensional model was smoothed by Geomagic Studio 12.0 to make it similar to an actual stomach *in vivo* and suitable for calculation. The constructed model was verified by automated detection using watershed transform from markers (Grau et al., 2004; Yan et al., 2006). Subsequently, Solidworks was used to construct gastrojejunal anastomosis and gastric separation. Finally, the following 3D CGJ and SPGJ models were established (Figure 3). The esophagus had a length of 100 mm, and an internal diameter of 24 mm. The length of the duodenum and jejunum from the pylorus to the anastomosis is about 35 cm. The length of the anastomosis is 45 mm, and the separation of the SPGJ model is about 20 mm from the lesser curvature of the stomach. This study was carried out following Hospital regulations, and all volunteers approved this study and provided written informed consent.

### The numerical method and boundary conditions

#### Food flow simulation

Fluid flow in the stomach is resumed water as a Newtonian Fluid. The numerical work was carried out based on the incompressible Navier-Stokes equation and the conservation of mass:

$$\rho \left[\frac{\partial u}{\partial t}+\left(u∙\nabla \right)u\right]+\nabla p-\mu \nabla^{2}u=0$$ (1)

∇∙u=0 (2)

where ***u*** is the fluid velocity vector, *p* is the pressure, *ρ* (*ρ* = 998.2 kg/m^3^) is the density, and *μ* is the viscosity (0.001003 kg/m s).

A steady flow (0.04 m/s) was imposed at the inlet (Acharya et al., 2021), and the outlet boundary was applied to the traction-free boundary condition. All the walls in the model were treated as non-slip rigid. The governing equations for food flow were solved in the computational fluid dynamics software ANSYS FLUENT CFD (ANSYS Inc., Canonsburg, PA). The solver adopted a SIMPLE algorithm of pressure-velocity interaction. The momentum equation was discretized in an upwind scheme with second-order precision. The models were constructed from unstructured grids, and especially refined grids were created for the near-wall regions to obtain the sharp change there. A mesh independency test was performed to evaluate computations and denser cases, which was achieved at the velocity field difference between two cases less than 5%. The convergence criterion of the residual is set to 10^–5^.

#### Food particle simulation

The discrete phase model (DPM) was used to calculate the movement of food particles. The equations of particle motion are given by

$$\frac{{du}_{p}}{dt}=F_{D}\left(u-u_{p}\right)+\frac{\mu \left(\rho_{p}-\rho \right)}{\rho_{p}}+F_{x}$$ (3)

where u is the fluid velocity, $u_{p}$ is the particle velocity. μ is the hydrodynamic viscosity, ρ is the fluid density (*ρ* = 998.2 kg/m^3^), $\rho_{p}$ is the particle density ( $\rho_{p}$ = 1300 kg/m^3^), and $d_{p}$ is the particle diameter ( $d_{p}$ = 50 nm). $F_{D}$ is the drag force per unit mass of the particle, which is given by

$$F_{D}=\frac{18\mu }{\rho_{p}d_{p}^{2}}\frac{C_{D}Re}{24}$$ (4)

where *C*_*D*_ is the drag coefficient. Re is Reynolds number (particle Reynolds number), which is given by

$$Re=\frac{\rho d_{p}\left|u_{p}-u\right|}{\mu }$$ (5)

The present study assumed that the particle was injected at the inlet. The inlet and outlet were set to the escape boundary. The particle-wall interaction boundary condition was assumed to be the elastic collision boundary. The equations of particle motion were solved in Fluent software. Semi-implicit trapezoidal integration is applied to solve the discrete phase motion equation, and the convergence criterion of the residual is set to 10^–5^.

## Results

### Clinical efficacy

A total of 73 patients were enrolled in the study, 48 underwent SPGJ, and 25 underwent CGJ. The baseline characteristics and preoperative nutritional status of the patients are shown in Table 2. For sex (*p* = 0.097), age (*p* = 0.618), BMI (*p* = 0.559), disease characteristics (*p* = 0.933), preoperative gastrointestinal decompression (*p* = 0.135), preoperative total parenteral nutrition (*p* = 0.173), total parenteral nutrition duration (*p* = 0.117), ascites (*p* = 0.999), hemoglobin (*p* = 0.490), serum albumin (*p* = 0.086), total lymphocyte (*p* = 0.274), total cholesterol (*p* = 0.617), CONUT score (*p* = 0.416), and preoperative nutritional status (*p* = 0.371) were comparable between the two groups.

**TABLE 2:** The baseline characteristics and preoperative nutritional status of the patients.

	SPGJ (n = 48)	CGJ (n = 25)	*p*-Value
Sex	36/12	14/11	0.097
Male/Female			
Age (y)	63.9 ± 10.3	65.2 ± 11.0	0.618
BMI (kg/m^2^)	21.8 ± 2.7	21.4 ± 3.1	0.559
Disease characteristics			0.933
Gastric cancer	38	20	
Benign pyloroduodenal obstruction	10	5	
Preoperative gastrointestinal decompression	28	19	0.135
Preoperative total parenteral nutrition	31	20	0.173
Total parenteral nutrition duration (d)	6.2 ± 2.4	7.5 ± 3.2	0.117
Ascites	8	4	0.999
Hemoglobin (g/L)	110.8 ± 19.9	107.5 ± 17.1	0.490
Serum albumin (g/L)	34.6 ± 3.9	32.8 ± 4.7	0.086
Total lymphocyte (10^a^ ^9^/L)	1.3 ± 0.7	1.5 ± 0.7	0.274
Total cholesterol (mmol/L)	3.8 ± 1.1	3.9 ± 1.2	0.617
CONUT assessment (score)			0.416
Normal (0-1)	0	0	
Light (2–4)	10	7	
Moderate (5–8)	32	13	
Severe (9–12)	6	5	
Preoperative nutritional status	17/27/4/0	5/18/2/0	0.371
GOOSS 0/1/2/3			

Values are presented as mean ± standard deviation or median (interquartile range).

^a^ Statistically significant values.

SPGJ, stomach-partitioning gastrojejunostomy; CGJ, conventional gastrojejunostomy; BMI, body mass index; CONUT, Controlling Nutritional Status; GOOSS, gastric outlet obstruction scoring system.

In terms of approach (*p* = 0.187), operative time (*p* = 0.141), and blood loss (*p* = 0.466), no significant between-group differences were observed. Compared with the SPGJ group, recovery of the CGJ group was slower in terms of time to pass gas (3 *versus* 4 days, *p* < 0.001), time to oral intake (3 *versus* 4 days, *p* = 0.001), and postoperative hospitalization (7 *versus* 9 days, *p* < 0.001). No anastomotic leakage occurred in both group after surgery. Anastomotic stenosis (CD-II) due to anastomotic edema occurred in one patient in the CGJ group, and recovered after conservative treatment. Anastomotic bleeding (CD-III) occurred in one patient in the SPGJ group and recovered after secondary surgery. Anastomotic bleeding (CD-II) occurred in two patients in the CGJ group and recovered after conservative treatment. In terms of short-term complications and CD grade (≤30 days), the SPGJ group was significantly better than the CGJ group. DGE occurred in 1 (2.1%) and 9 patients (36%) of the SPGJ and CGJ groups, respectively (*p* < 0.001). Based on the ISGPS grading standard, 4 patients (16%) had grade A DGE, 3 patients (12%) had grade B, and 2 patients (8%) had grade C in the CGJ group. One patient in the CGJ group was discharged after endoscopic jejunal feeding tube placement (CD-Ⅲ) due to severe DGE (grade C), and two were discharged after gastric tube retention. 1 patient (2.1%) had grade A DGE in the SPGJ group. No mortality within 30 days of surgery was observed in both groups. SPGJ group had advantages over the CGJ group in terms of postoperative nutritional status (*p* < 0.001). Based on the GOOSS, 5 patients (10.4%) had GOOSS 2, and 43 patients (89.6%) had GOOSS 3 in the SPGJ group. 3 patients (12%) had GOOSS 0, 4 patients (16%) had GOOSS 1, 8 patients (32%) had GOOSS 2, and 10 patients (40%) had GOOSS 3 in the CGJ group.

### Numerical results

**FIGURE 4:**
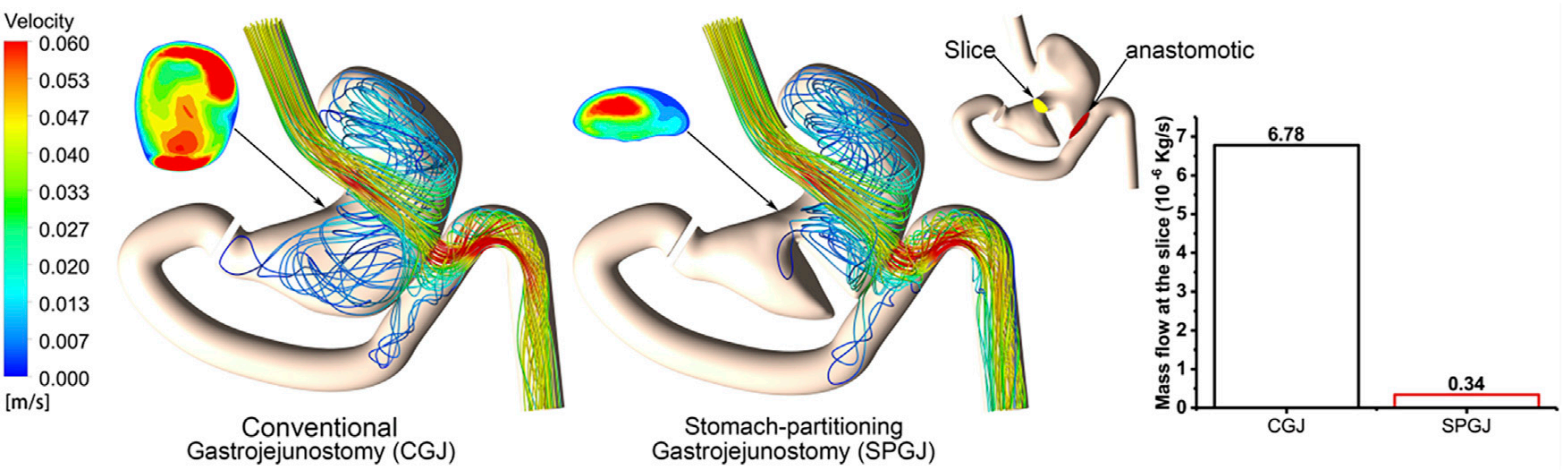
The streamlined distribution and flow at different locations of CGJ and SPGJ were compared.

Figure 4 shows the internal gastric streamline under different surgical procedures. Contents in the SPGJ model are blocked by the separating surface, and most of them flow out directly through the anastomosis with almost no fluid flow occurring near the pylorus. However, obvious complex flow performance is observed in the CGJ model near the pylorus. Moreover, the flow in the upper part of the stomach in the SPGJ model is more complicated than that in the CGJ model. The SPGJ model causes a remarkable long flow recirculation area near the strut in the gut near the anastomotic stoma. To quantitatively analyze the blocking impact of SPGJ, the fluid flows to the pylorus are calculated. As evident from histograms (Figure 4), the content flow into the pylorus is only 5% of that in the CGJ model.

**FIGURE 5:**
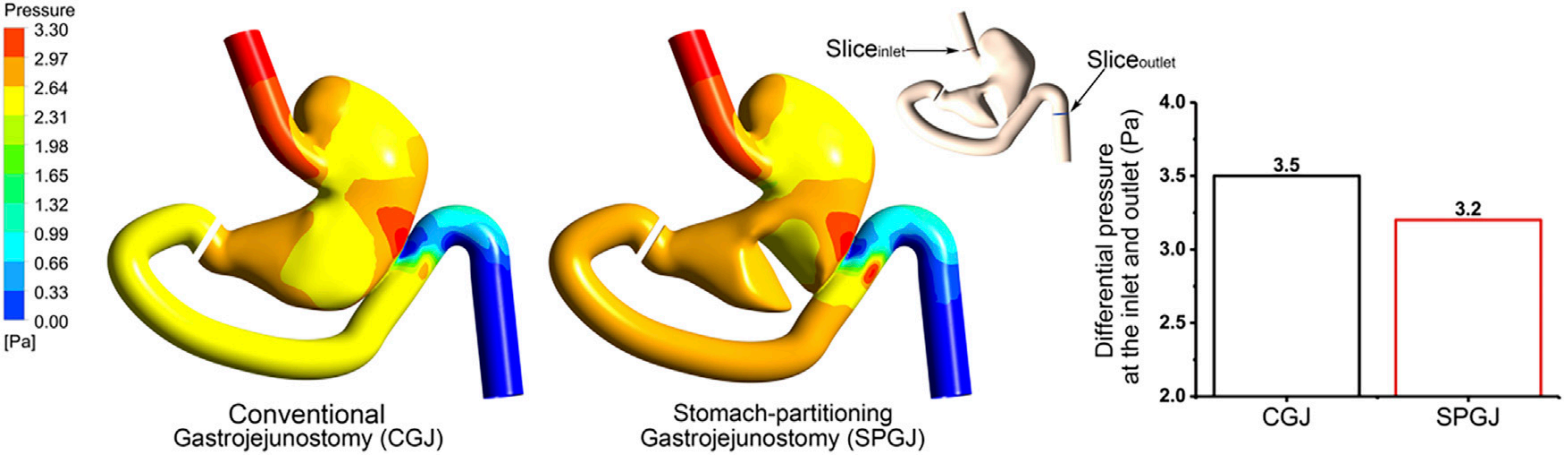
The pressure distribution and inlet and outlet pressure differences of CGJ and SPGJ were compared.

Figure 5 shows different pressure distributions in the stomach under different surgical procedures. Compared with the CGJ model, the SPGJ model decreases the pressure in the upper stomach, while it leads to significantly a high level of pressure at the distal gastric and input loop intestinal. To quantitatively demonstrate difference between the two models, the pressure drop between the outlet and inlet of the digestive system is calculated. Histograms in Figure 5 reveal that the pressure drop in the SPGJ model is reduced by 9% compared with the CGJ model.

**FIGURE 6:**
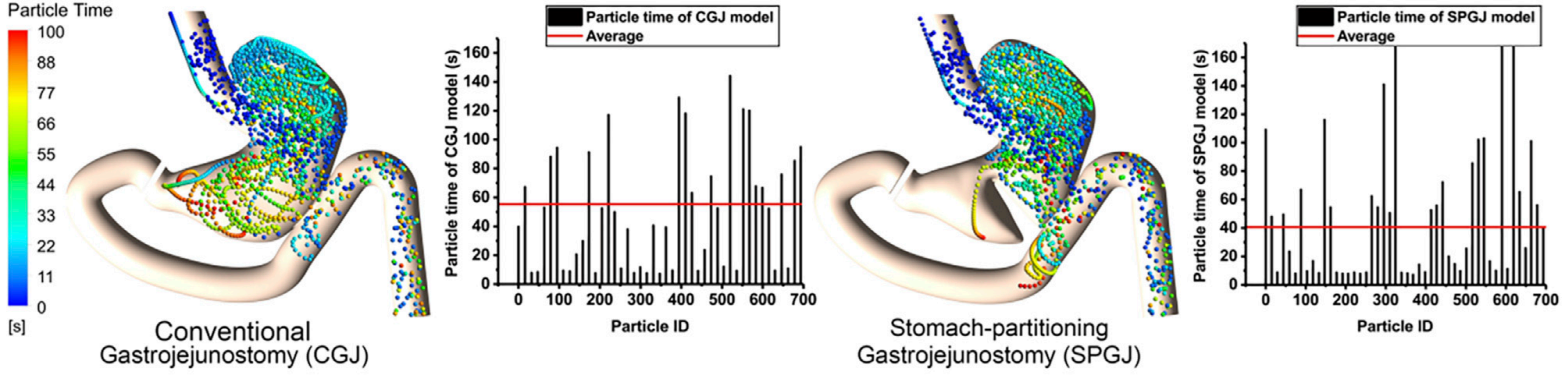
The particle retention time of CGJ and SPGJ were compared.

Figure 6 shows the instantaneous spatial distribution of discrete phase particles in the stomach after different surgical procedures. Two treatments show different retention times in the stomach, and the shorter retention time of particles is observed in the SPGJ model. Some particles with a longer retention time are observed in the middle of the stomach and the pylorus in the CGJ model, while there were almost none in the SPGJ model. Besides, we found more long retention particles at the jejunum. To quantitatively analyze particle retention in the two models, the retention time of every particle is calculated. We found that the retention time of most particles in the CGJ model was higher than that in the SPGJ model, and the average retention time of particles in the CGJ model is 1.5 times longer that in the SPGJ model.

**FIGURE 7:**
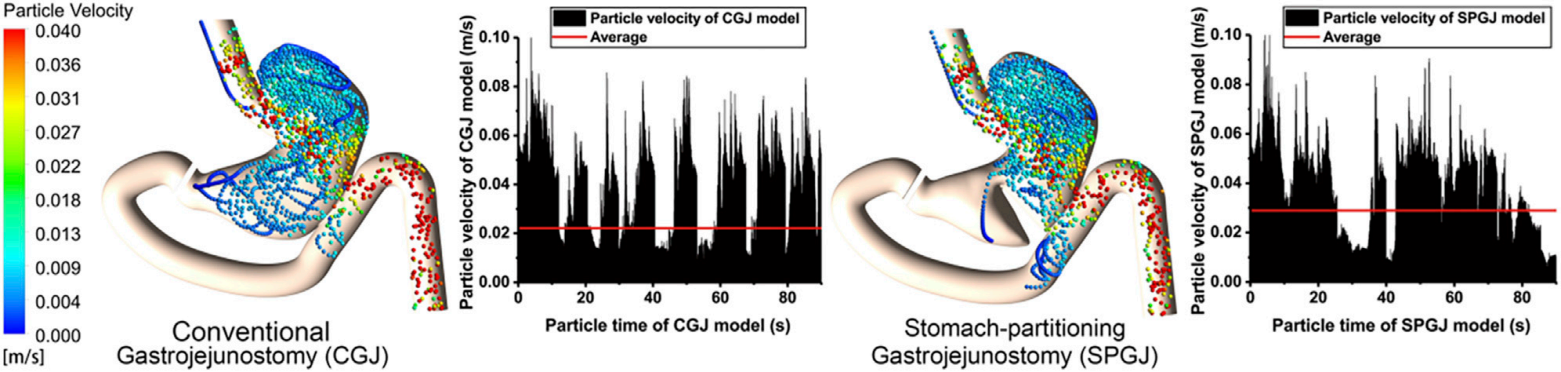
The instantaneous velocity of discrete phase particles of CGJ and SPGJ were compared.

Figure 7 shows the instantaneous velocity of discrete phase particles in the stomach after different surgical procedures. Compared with the CGJ model, particles in most region of the SPGJ model has a high level of velocity, but very low near the pylorus. The average velocity of particles in the CGJ model is 22 mm/s, while it is 29 mm/s in the SPGJ model. Besides, the SPGJ model leads to more particles with low velocity accumulated in the upstream of the jejunum.

## Discussion

At present, some scholars have compared the incidence of DGE between CGJ and SPGJ through meta-analysis. However, the DGE evaluation criteria in the included studies are not uniform, so it remains controversial (Kumagai et al., 2016; Lorusso et al., 2019). In our study, the ISGPS classification is widely used at present, which can effectively avoid the bias caused by subjective classification. Our results showed that the SPGJ group had a significant advantage over the CGJ group in the incidence and grade of postoperative DGE, and it did not increase the operation time and anastomotic complications. The existing studies have not analyzed the reasons for the high incidence of DGE after CGJ and the low incidence of DGE after SPGJ. Combined with clinical analysis, we think the reason for the high incidence of DGE after CGJ surgery is that gastric contents always preferentially flow to the pyloric rather than the anastomosis. However, the separation of SPGJ changes the flow direction of stomach contents, making it easier to flow to the anastomosis.

To prove our idea, we conducted numerical simulations through 3D models of CGJ and SPGJ. Since the short survival period of GOO patients with advanced tumors, digesting or absorbing food without simulating tumor growth after surgery is the main problem to solve. This numerical study found that the SPGJ model increase fluid flow in most region of the stomach, but suppress flow near the pylorus and pressure drop, which would decrease the resistance of the stomach to foods and help with gastric emptying. We also get this conclusion by analyzing low particle retention time and high particle velocity in the SPGJ model. Hence, the SPGJ model may have a better performance than CGJ in terms of gastric emptying and tumor protection, which is consistent with our clinical conclusion. Furthermore, we found abnormal high pressure distributed at the partitioning surface and anastomotic stoma, and a portion of gastric contents in the upper jejunum below the anastomosis would be countercurrent to the duodenum. Although normal peristalsis of the intestine can drive these gastric contents to the distal jejunum, this may reduce the efficiency of gastric emptying.

As to whether the position relationship between gastrointestinal anastomosis and transverse colon in the two surgical procedures affects the postoperative efficacy, there is some controversy in the previous literature (Kaminishi et al., 1997; Kumagai et al., 2016). In our study, gastrojejunostomy was performed mostly behind the transverse colon in both groups. Its advantage is that the input loop is shorter, conducive to gastric emptying, and anastomosis was almost unaffected by the change of position. At present, in the field of digestive systems with irregular luminal peristalsis and strong expansibility, it is difficult to construct an actual stomach model and carry out hydrodynamics analysis. We first applied the 3D model to the flow simulation of digestive tract reconstruction after surgery and compared the gastric emptying performance of SPGJ and CGJ. In the future, a 3D model combined with numerical simulation may become a powerful tool for the study of digestive diseases, to help clinicians further understand the biomechanical mechanism of the digestive tract in normal and pathological conditions.

There were some limitations in our study. The present study assumed a rigid wall without performing fluid-structure interaction simulations, which might have little impact on the results. The major conclusion would not be influenced by the stomach wall moving during the gastric filling phase. The interactions and chemical reactions between particles and particle-stomach were also not considered in the numerical simulation. The idealized flat inlet flow could be another limitation of this study. However, it is difficult to measure speed during eating, and no document report this for different groups. Additionally, the sensitivity analysis due to the variability of stomach geometry is important and lacking for these numerical results, and more patients’ stomach models should be constructed to verify our conclusion. Finally, it was a retrospective study with small sample size (Table 3).

**TABLE 3:** The surgical outcomes and postoperative outcomes of the patients.

	SPGJ (n = 48)	CGJ (n = 25)	*p*-Value
Approach			0.187
Total laparoscopy	42	18	
Laparotomy	6	7	
Operative time (min)	125 (105–160)	155 (99–188)	0.141
Blood loss (ml)	20 (20–50)	50 (15–50)	0.466
Time to pass gas (d)	3 (2–4)	4 (3–5)	<0.001^a^
Time to oral intake (d)	3 (2–3)	4 (3–4)	0.001^a^
Postoperative hospitalization (d)	7 (6–8)	9 (8–13)	<0.001^a^
complications (≤ 30 days)	2	12	<0.001^a^
Anastomotic leakage	0	0	0.999
Anastomotic stenosis	0	1	0.342
Anastomotic bleeding	1	2	0.557
Delayed gastric emptying	1 (2.1%)	9 (36%)	<0.001^a^
Grade A/B/C	1/0/0	4/3/2	<0.001^a^
CD grade I/II/III/IV	0/1/1/0	0/11/1/0	<0.001^a^
Mortality	0	0	0.999
Postoperative nutritional status	0/0/5/43	3/4/8/10	<0.001^a^
GOOSS 0/1/2/3			

Values are presented as mean ± standard deviation or median (interquartile range).

^a^ Statistically significant values.

SPGJ, stomach-partitioning gastrojejunostomy; CGJ, conventional gastrojejunostomy; CD, Clavien-Dindo; GOOSS, gastric outlet obstruction scoring system.

## Conclusion

In summary, our results proved that compared with CGJ, patients after SPGJ had better gastric emptying performance, gastric contents were more conducive to discharge to the anastomosis, and postoperative clinical efficacy was better. Therefore, we think that SPGJ may be a better option for treating GOO, which is beneficial to improve the patient’s quality of life after surgery.

## Data Availability

The raw data supporting the conclusions of this article will be made available by the authors, without undue reservation.

## Abbreviations

GOO, gastric outlet obstruction; DGE, delayed gastric emptying; CGJ, conventional gastrojejunostomy; SPGJ, stomach-partitioning gastrojejunostomy; 3D, three-dimensional; CT, computed tomography; GOOSS, gastric outlet obstruction scoring system; COUNT, controlling nutritional status; CD, clavien-dindo; DPM, discrete phase model.
