# Supplementary material for: Gastric emptying performance of stomach-partitioning gastrojejunostomy versus conventional gastrojejunostomy for treating gastric outlet obstruction: A retrospective clinical and numerical simulation study
Source: Front Bioeng Biotechnol. 2023 Feb 17;11:1109295. doi: 10.3389/fbioe.2023.1109295 (PMC9982392; doi:10.3389/fbioe.2023.1109295)
Supplement: Supplementary file 1 [file DataSheet1.PDF]

首都医科大学附属北京友谊医院

生命伦理委员会伦理审查批件

批件号：2022-P2-210-01

试验项目名称	分隔式胃空肠吻合与传统胃空肠吻合治疗晚期胃癌伴胃出口梗阻的安全性及疗效对比分析				
申办单位	首都医科大学附属北京友谊医院				
项目来源	自筹				
本院申请科室	普外科	承担责任	□组长单位<br>□参加单位<br>√单中心	项目负责人	张军
主要审查文件	1.递交信,-/-； 2.初始审查申请,-/-； 3.本中心研究项目负责人简历及执业证书、职称证书、GCP 培训证书,-/-；4.本中心项目负责人的利益冲突声明和保密承诺,-/-； 5.临床研究方案,v1.0/2022-06-16； 6.免除知情同意申请,-/-；				
伦理委员会声明	*本伦理委员会严格按照 ICH/GCP、中国 GCP 及相关法规组成和工作。<br>*本伦理委员会的组成和工作相对独立。				
审查方式	□ 会议审查 √ 快速审查 √ 初始审查 □ 跟踪审查 复审				
审查时间	2022-06-30	会议地点	不适用		
审查委员	薛富善,武珊珊				

审查意见：

根据中共中央办公厅、国务院办公厅《关于深化审评审批制度改革鼓励药品医疗器械创新的意见》(2017)的伦理原则，国家卫计委《涉及人的生物生命研究伦理审查办法》(2016)，国家药品监督管理局、国家卫生健康委员会《药物临床试验质量管理规范》(2020)，国家卫计委《医疗器械临床试验质量管理规范》(2016)，WMA《赫尔辛基宣言》和 CIOMS《人体生物生命研究国际道德指南》的指导原则，经本伦理委员会审查**同意按所批准的临床研究方案等开展本项研究。**

备注：

1. 如涉及中国人类遗传资源行政许可或备案，在取得中国人类遗传资源管理办公室批件或完成中国人类遗传资源备案后方可依法依规开展研究。
2. 本项临床试验应当在伦理委员会同意进行之日起 1 年内实施。逾期未实施的，本批件自行废止。
3. 修正后同意/复审项目，应将修正后文件及时反馈给伦理委员会，以便签署意见或安排复审。
4. 在试验实施过程中，如需对研究方案、知情同意书等文件做任何修改，应及时向本伦理委员会提交修改申请，经重新审查，获得批准后方可执行。
5. 发生严重不良事件及可能影响风险/受益比的任何事件和新信息须及时报告伦理委员会。
6. 定期/年度跟踪审查项目，于到期前 1 个月内（无论试验开始与否）提交定期跟踪审查申请。
7. 如有不依从/违背方案或暂停/提前终止的试验项目，应及时以书面文件告知本伦理委员会。

8. 临床试验结束后，须及时向伦理委员会提交结题报告。	
年度/定期跟踪审查频率	☐ 3 个月 ☐ 6 个月 √ 12 个月 ☐ 其他 ______
有效期	12 个月
主任委员/副主任委员签字：<br>伦理委员会（盖章）<br>日 期：2022 年 07 月 05 日	

伦理委员会地址：北京市西城区永安路 95 号（邮编：100050） 联系人：李悦 电话/传真：010-63139006
